# Generation and Genetic Stability of a PolX and 5′ MGF-Deficient African Swine Fever Virus Mutant for Vaccine Development

**DOI:** 10.3390/vaccines12101125

**Published:** 2024-09-30

**Authors:** Daniel Pérez-Núñez, Daniel W. Madden, Gonzalo Vigara-Astillero, David A. Meekins, Chester D. McDowell, Bianca Libanori-Artiaga, Raquel García-Belmonte, Dashzeveg Bold, Jessie D. Trujillo, Konner Cool, Taeyong Kwon, Velmurugan Balaraman, Igor Morozov, Natasha N. Gaudreault, Yolanda Revilla, Juergen A. Richt

**Affiliations:** 1 Microbes in Health and Welfare Department, Centro de Biología Molecular Severo Ochoa (CBM), CSIC-UAM, c/ Nicolás Cabrera 1, 28049 Madrid, Spain; daniel_perez@cbm.csic.es (D.P.-N.); gvigara@cbm.csic.es (G.V.-A.); raquel.g.b@cbm.csic.es (R.G.-B.); 2 Center of Excellence for Emerging and Zoonotic Animal Diseases, Department of Diagnostic Medicine/Pathobiology, College of Veterinary Medicine, Kansas State University, 1800 Denison Ave, Manhattan, KS 66506, USA; dwmadden@vet.k-state.edu (D.W.M.); dmeekins@k-state.edu (D.A.M.); cdmcdow@vet.k-state.edu (C.D.M.); blartiaga@vet.k-state.edu (B.L.-A.); bold@vet.k-state.edu (D.B.); jdtrujillo@vet.k-state.edu (J.D.T.); konnerc@vet.k-state.edu (K.C.); tykwon@vet.k-state.edu (T.K.); balarama@vet.k-state.edu (V.B.); imorozov@vet.k-state.edu (I.M.); nng5757@vet.k-state.edu (N.N.G.)

**Keywords:** ASFV, vaccine, Arm/07/CBM/c2, PolX gene, MGF genes, NGS, viral populations, COS-1, LAV

## Abstract

The African swine fever virus (ASFV) causes fatal disease in pigs and is currently spreading globally. Commercially safe vaccines are urgently required. Aiming to generate a novel live attenuated vaccine (LAV), a recombinant ASFV was generated by deleting the viral O174L (PolX) gene. However, during in vitro generation, an additional spontaneous deletion of genes belonging to the multigene families (MGF) occurred, creating a mixture of two viruses, namely, Arm-ΔPolX and Arm-ΔPolX-ΔMGF. This mixture was used to inoculate pigs in a low and high dose to assess the viral dynamics of both populations in vivo. Although the Arm-ΔPolX population was a much lower proportion of the inoculum, in the high-dose immunized animals, it was the only resulting viral population, while Arm-ΔPolX-ΔMGF only appeared in low-dose immunized animals, revealing the role of deleted MGFs in ASFV fitness in vivo. Furthermore, animals in the low-dose group survived inoculation, whereas animals in the high-dose group died, suggesting that the lack of MGF and PolX genes, and not the PolX gene alone, led to attenuation. The two recombinant viruses were individually isolated and inoculated into piglets, confirming this hypothesis. However, immunization with the Arm-ΔPolX-ΔMGF virus did not induce protection against challenge with the virulent parental ASFV strain. This study demonstrates that deletion of the PolX gene alone neither leads to attenuation nor induces an increased mutation rate in vivo.

## 1. Introduction

African swine fever virus (ASFV), the only member of the family *Asfarviridae* [1], is the etiological agent of African swine fever (ASF), a severe disease affecting both wild boar and domestic pigs that produces up to 100% mortality. ASFV was first identified in Africa in 1921 and spread to Europe in the 1950s and to the Caucasus region in 2007. Subsequent to its introduction to the Caucasus, the virus continued to spread, and it currently affects more than 40 countries in Europe, Oceania, and Asia [2]. Currently, it is endemic in China, the world’s largest pork producer, and affects neighboring countries such as Vietnam, Laos, Myanmar, Korea, and the Philippines. It recently emerged for the first time in many decades in the Americas, the Dominican Republic, and Haiti [3]. The widespread emergence of ASFV in major pork-producing countries has resulted in a destabilization of the food supply chain, making ASFV one of the most important animal health concerns worldwide. The development of safe and efficacious vaccines against ASFV is urgently needed to address the threat to worldwide pork production and the food supply.

Several live attenuated vaccine (LAV) candidates for ASF have been described; however, the level of protection induced by these LAV candidates is variable, especially against heterologous ASFV strains, and they can have poor side effect profiles in vaccinated animals [4,5,6,7,8,9,10,11,12,13], as reviewed in [14]. While the development of LAVs is a promising strategy for ASF vaccine development, the successful deployment of ASF LAV candidates faces multiple challenges, including the lack of stable cell lines for ASFV propagation, the potential for residual virulence and inadequate vaccine safety profiles, an inability to differentiate infected from vaccinated animals (DIVA) associated with current LAV candidates [15,16], and a lack of extensive genetic characterization and analysis by next-generation sequencing (NGS) to evaluate and confirm the genetic stability of prototype LAV vaccine strains [5,12,13].

The ASFV O174L gene encodes the viral DNA polymerase PolX [17], a unique reparative DNA polymerase with similarities to the X family of β DNA polymerases which is located in the extracellular viral particle [18]. ASFV PolX is involved in base excision repair (BER) of viral DNA [19], together with other ASFV proteins such as those encoded by the ASFV genes E296R, NP429L, E301R, and D345L [20]. Additionally, ASFV PolX has been shown to be involved in the repair of mutated viral DNA resulting from oxidative damage during ASFV replication in primary porcine alveolar macrophages (PAMs). PolX has been shown to be dispensable for ASFV replication in Vero cells but essential for replication in PAMs [21]. We hypothesized that a recombinant ASFV lacking the O174L gene would accumulate deleterious mutations during replication, resulting in attenuation of the virus in vivo.

Here, we present studies performed with a recombinant ASF virus derived from the virulent genotype II Arm/07 (Arm/07/CBM/c2) ASFV isolate [22] in which we have deleted the O174L gene (designated Arm-ΔPolX) using CRISPR-Cas9 technology in COS-1 cells, as previously described [11]. While no off-target mutations were found in terms of indels or SNPs compared to the parental Arm/07/CBM/c2 virus, an unexpected 26 kb deletion was identified within the 5′ end of the viral genome, resulting in a recombinant virus designated Arm-ΔPolX-ΔMGF. The 5′ region of the ASFV genome harbors several genes belonging to the multigene families (MGFs), which have been recognized to play a role in modulating host immune responses [9,23,24,25,26]. Some of these genes have been related to the ability to infect PAMs [26] and in the control of type I IFN [23]. In particular, MGF110-9 has been involved in controlling TBK1 degradation [27], MGF505-7R in impairing cGAS-STING pathway regulation and JAK-mediated signaling [28,29] and MGF360-1L in inducing JAK1 degradation [30]. Indeed, deletion of some of these genes results in the attenuation of virulent strains, which in turn results in protection against virulent strains, including several members of MGF505 and MGF360 [9,12]. However, not all MGF genes are essential for virulence since the deletion of MGF360-1L [31], MGF110-5L-6L [32], or MGF110-1L [33] from a virulent strain did not lead to the attenuation in vivo. On the other hand, deletion of several MGF genes can lead to attenuation of certain prototypes but still not achieve adequate levels of protection after immunization [34]. Although the function of many of these genes has been described and studied, especially in relation to virulence and virus–host interaction, the function of many of them remains unknown.

A mixed population of Arm-ΔPolX and Arm-ΔPolX-ΔMGF viruses was used to test the viral population dynamic in vivo in a high- and low-dose scenario. Despite being a much lower proportion of the total viral population in the mixed inoculum, the Arm-ΔPolX virus was able to overgrow the Arm-ΔPolX-ΔMGF mutant virus in the high-dose immunized animals, and all of these animals died. In contrast, animals immunized at a low dose with the mixed inoculum survived, with the Arm-ΔPolX-ΔMGF virus predominating, indicating that the Arm-ΔPolX maintained its virulence, whereas the Arm-ΔPolX-ΔMGF virus was attenuated. Using purified Arm-ΔPolX or Arm-ΔPolX-ΔMGF viruses confirmed that a deletion of the viral PolX alone is insufficient to attenuate virulent ASFV and that Arm-ΔPolX-ΔMGF is attenuated in vivo. Interestingly, immunization of animals with Arm-ΔPolX-ΔMGF failed to induce protection against challenge with the virulent parental Arm/07 strain. Further experiments are necessary to evaluate the protective effect of Arm-ΔPolX-ΔMGF at higher doses and whether deletion of PolX may enhance the attenuation and safety of ΔMGF ASFV LAVs.

## 2. Materials and Methods

### 2.1. Cells and Viruses

The hemadsorbing viral isolate Arm/07/CBM/c2, a genotype II ASFV strain, was isolated from infected domestic pigs during an ASFV epizootic not purified and amplified through cell line passages, as described previously [22], maintaining its virulence in vivo [35].

COS-1 cells derived from African green monkeys and obtained from the ATCC (American Type Culture Collection, Manassas, VA, USA) were cultured in Dulbecco’s Modified Eagle Medium (DMEM; Corning, Corning, NY, USA) with 5% fetal bovine serum (FBS; Sigma-Aldrich, St. Louis, MO, USA) and supplemented with 2 mM L-glutamine, 100 U/mL gentamycin, and 0.4 mM non-essential amino acids. Porcine alveolar macrophages (PAMs) were extracted from pig bronchoalveolar lavage, as described previously [36]. PAMs were cultured on DMEM with 10% pig serum (Sigma-Aldrich) supplemented as described above. Cells were grown at 37 °C in a saturated water vapor atmosphere with 5% CO_2_.

### 2.2. Plasmids and Cloning

Two different vectors were used for the generation of the recombinant Arm07∆PolX-GFP virus using CRISPR-Cas9 technology: (i) pSpCas9(BB)-2A-Puro (PX459), in which the Nuclear Localization Signal (NLS) has been deleted (gifted by Bruno Hernaez), and (ii) pcDNA3.1-derived donor vector containing the flanking sequences of the target gene (O174L) surrounding a EGFP fluorescent marker gene derived from the pEGFP-E3 vector under a viral promoter.

Specific gRNAs were cloned into the pSpCas9(BB)ΔNLS-2A-Puro vector to disrupt the O174L gene. gRNAs were designed by the Protospacer workbench based on the O174L sequence of the ASFV strain Georgia 2007/01 (FR682468.1), which is 99.992% sequence identity to Arm/07/CBM/c2. The designed gRNA sequences were as follows: 5′ TCTTTTTACGGCCTTAGCCGAGG 3′ (gRNA0) and 5′ TTTTCAGTAGTGAT-TTTTAGAGG 3′ (gRNA1), generating the vectors pSpCas9(BB)ΔNLS-2A-Puro_O174L-gRNA-0 and pSpCas9(BB)ΔNLS-2A-Puro_O174L-gRNA-1.

The pcDNA3.1 vector (Invitrogen, Waltham, MA, USA) was used as a backbone for the generation of the donor vector. The O174L gene (basepair (bp) 129,000–129,524 of the ASFV Arm/07/CBM/c2 genome, PRJEB38146) and its flanking regions (500 bp upstream and 500 bp downstream) were first cloned (pFL-O174L), and then the O174L gene was replaced with the EGFP gene derived from an pEGFP-E3 vector (pFL-ΔO174L-GFP). Finally, we replaced the CMV promoter of the EGFP gene with the ASFV p72 promoter (bp 105,530–105,570 of the ASFV Georgia 2007/01 genome, FR682468.1) [37], generating the final donor vector pFL-ΔO174L-p72GFP.

The specific probes designed for cloning by In-Fusion (Takara Bio) technology were as follows: 5′-CCA GAT ATA CGC GTT GCC TTT GAA GAC GCT GTG C-3′, 5′-TTT CCG CCT CAG AAG TCT CCA GTC CAG GTC TTG ACA-3′, and 5′-AAC GCG TAT ATC TGG CCC G-3′, 5′-CTT CTG AGG CGG AAA GAA CCA-3′ for pFL-O174L vector; 5′-GCT TGT TTT GAG CAA ATT GTT TAA GCA A-3′, 5′-TTT ATA TTT AAT ATT AAA ATC TTT TCA TTT TAT ATA TTA TAT ACG CAA AAT GG-3′, and 5′-TTG CTC AAA ACA AGC GAC ATT GAT TAT TGA CTA GTT ATT AAT AGT AAT CAA TTA CG-3′, 5′-AAT ATT AAA TAT AAA CCA TAG AGC CCA CCG C-3′ to substitute the O174L gene by EGFP under CMV promoter. For p72 promoter cloning, we designed the specific probes: 5′-TGG AGT TCC GTA TTT AAT AAA AAC AAT AAA TTA TTT TTA TAA CAT TAT ATA GGT CGC CAC C-3′, 5′-GGT GGC GAC CTA TAT AAT GTT ATA AAA ATA ATT TAT TGT TTT TAT TAA ATA CGG AAC TCC A-3′, and 5′-GGT CGC CAC CAT GGT GAG-3′, 5′-CGG AAC TCC ATA TAT GGG CTA TG-3′. The p72 promoter was generated by incubation of 10 µL at 100 µM of each of the specific probes in H_2_O (final volume 100 µL), incubating 5 min at 95 °C and 30 min at 25 °C. This product was cloned into the pFL-ΔO174L-GFP by In-Fusion technology, generating the final donor vector, pFL-ΔO174L-p72GFP.

### 2.3. Generation of Recombinant Virus Arm∆PolX by CRISPR-Cas9

The recombinant Arm∆PolX virus was generated in COS-1 cells by CRISPR-Cas9 technology, as previously described [11]. Briefly, COS-1 cells were co-transfected with specific pSpCas9(BB)ΔNLS-2A-Puro gRNAs together with the donor vector pFL-ΔO174L-p72GFP using FuGene HD (Promega, Madison, WI, USA). At 24 h post transfection (hpt), 1 µg/mL puromycin (Sigma-Aldrich) was added to the media of cells. After an additional 24 h, transfected cells were infected at two different MOIs (1 and 0.1) with the ASFV isolate Arm/07/CBM/c2. Following 1.5 h of viral adsorption, 1 µg/mL puromycin was added to the medium. At 5 days post-infection (dpi), cells and media containing the viral progeny were collected and stored at −80 °C.

### 2.4. Plaque Isolation of Recombinant Viruses and Viral Growth Assessment

Collected recombinant viruses were used to infect COS-1 cells. After viral adsorption, inoculum was removed and DMEM-1% agar was added. At 4–7 days post-infection (dpi), recombinant plaques corresponding to ΔPolX viruses were detected under fluorescent microscopy. Recombinant plaques were collected by sterile pipette tips in 40 µL of DMEM and stored at −80 °C. After 3 freeze/thaw cycles, the collected virus was used to infect new COS-1 cells using the same procedure described above. This plaque isolation method was repeated at least five times to separate recombinant virus from wild-type virus.

During the isolation procedure, the presence of wild-type contaminant virus was tested by PCR. Then, 10 µL of the isolated plaque was digested with proteinase K (Sigma-Aldrich) in 1.5 mM MgCl_2_, 50 mM KCl, 0.45% Tween20, 0.45% NP40, and 10mM Tris-HCl pH 8.3 buffer and incubated for 30 min at 45 °C and 15 min at 95 °C. The digested, isolated plaque was used as a DNA template for PCR to detect the presence of recombinant or wild-type parental virus. The primers used for the detection of recombinant and parental viruses by PCR were as follows: 5′-TTGCTCAAAACAAGCGACATTGATTATTGACTAGTTATTAATAGTAATCAATTACGG-3′ and 5′-AATATTAAATATAAACCATAGAGCCCACCGC-3′ for GFP detection and 5′-CGTTTCTTAGGTATGCGATACG-3′ and 5′-ATTGTAAATGACTTACGCTCCC-3′ for O174L gene detection.

For viral growth assessment, two 24-well plates seeded with PAMs at a density of 0.8 × 10^6^ cells/well and COS-1 cells at a density of 0.5 × 10^6^ cells/well were infected with Arm/07/CBM/c2 wild-type and Arm-∆PolX-ΔMGF virus at MOI = 2. After 2 h of adsorption at 37 °C, the viral inoculum was discarded, cells were washed twice with PBS, and DMEM containing 10% pig serum was added. Total cell lysates were obtained at 0, 24, 48, 72, and 96 h post-infection (hpi). At each time point, cells were collected, subjected to 3 freeze/thaw cycles, and titrated by hemadsorption (HAD) assays. For HAD assays, individual 60-well microtest plates (Greiner, Kremsmünster, Austria) were seeded with PAMs, and 5 µL of virus dilution was added to each well. After 16 h of infection, a drop of a solution of fresh pig erythrocytes was added to every well, and hemadsorption was assessed 96 h later.

### 2.5. In Vivo Animal Experiments

For initial safety studies of the deletion mutants, 10 crossbred piglets 3–4 weeks of age (mixed sex) were separated into two groups and housed in different pens at opposite ends of a single biocontainment room designed for housing large animals. One group of five pigs were inoculated intramuscularly (IM) with 1 mL of a 10^4^ plaque-forming unit (pfu) dose of the mixed Arm∆PolX/Arm∆PolX-∆MGF, and the other five pigs were inoculated IM with a 10^2^ pfu dose of the viruses. All handling of animals was performed first for the 10^2^ pfu dose pen, followed by decontamination before handling piglets administered the 10^4^ pfu dose to avoid cross-contamination. Observations for clinical signs, including body temperature checks, were performed daily to detect signs of ASF. Clinical scores, ranging from 0 (no clinical signs) to 3 (severe clinical signs), were collected for several parameters, including fever, liveliness, skin color, body shape, and respiratory/digestive issues. Blood collection was performed on days 0, 1, 3, 5, 7, 10, and 14 post-vaccination (DPV) or if a pig was humanely euthanized. Blood was collected in serum and EDTA tubes (BD, Franklin Lakes, NJ, USA) and stored at −80 °C for further analysis. Post-mortem analyses were performed for pigs found dead, humanely euthanized, and for all piglets at the end of the study period of 14 days post challenge (DPC). All work involving animals was performed under Select Agent guidelines within the biosafety level 3 (BSL-3Ag) facilities at the Biosecurity Research Institute at Kansas State University. Animal research was conducted in compliance with the Animal Welfare Act and other federal statutes and regulations relating to animal care and experimentation under protocol #3758, approved by the Institutional Animal Care and Use Committee (IACUC) at Kansas State University on 6 October 2016.

Following the identification and purification of both Arm∆PolX and Arm∆PolX-∆MGF viral populations, an additional safety and efficacy study was performed. Eighteen ASFV-naïve conventional outbred piglets aged 5–6 weeks were divided into three groups of 6 piglets each. Piglets in the first group were inoculated intramuscularly (IM) with 10^2^ HAD50/mL of ASFV vaccine strain Arm-ΔPolX, while piglets in the second group were given 10^2^ HAD50/mL of vaccine strain Arm-ΔPolX-ΔMGF IM, with the third group serving as unvaccinated controls. At 28 days post-vaccination (DPV), all vaccinated and unvaccinated piglets were challenged with 10^2^ HAD50/mL wild-type Arm/07 ASFV IM, with planned necropsies scheduled for 14 days post-challenge (DPC). Anticoagulated (EDTA) whole blood was collected by jugular venipuncture throughout this study at multiple timepoints post-vaccination and post-challenge to assess viral DNA levels. Piglets were monitored daily after both vaccination and wild-type challenge and assigned a total clinical score based on assessment of 8 clinical symptoms, including fever (0–4), liveliness (0–3), body shape (0–3), respiratory function (0–3), neurological signs (0–3), skin lesions (0–3), ocular/nasal discharge (0–3), and digestive signs (0–3), with a score of 0 indicating normal. Moribund piglets and those with a clinical score > 10 were humanely euthanized via intravenous pentobarbital administration. Following death, piglets were necropsied for pathological evaluation and tissue collection. Animal work and sample collection were performed at the Biosecurity Research Institute using protocols evaluated and approved by the Kansas State University Institutional Biosafety Committee (IBC; protocol #1314) and IACUC (protocol #4265). All animals were sourced from Oak Hill Genetics (USDA #33-A-0392) and tested negative for PRRSV, PCV, and SIV.

### 2.6. Viral DNA Extraction

Viral DNA extraction was performed as previously described [22]. Briefly, recombinant ASFV virus was grown in six P150 dishes of COS-1 cells, and supernatants were collected at 3 days post-infection (dpi). Supernatants were centrifuged overnight at 8281× *g* at 4 °C, and viral pellets were resuspended in a cold-filtered 10 mM Tris-HCl solution equilibrated at pH 8.8. Virus suspensions were treated with 0.25 U/mL DNAse I (Sigma-Aldrich), 0.25 U/µL S7 Nuclease (Sigma-Aldrich), and 20 µL/mL RNAse A (Promega) in a buffer containing 800 mM Tris-HCl (pH 7.5), 200 mM NaCl, 20 mM CaCl_2_, and 120 mM MgCl_2_ for 2 h at 37 °C. Following this, viral suspension was treated with 200 µg/mL proteinase K (Sigma-Aldrich) in 0.5% SDS solution for 1 h at 45 °C, and viral DNA was precipitated by incubation with a 1:1 ratio of phenol:chloroform:isoamyl alcohol (25:24:1). The solution was centrifuged at 9400× *g* for 3 min at room temperature, and the aqueous fraction was transferred and incubated with 0.1 volume of 3M acetic acid (pH 5.2), 1 µL lineal polyacrylamide (LPA; Sigma-Aldrich), and 2 volumes of cold absolute ethanol (100%) at −80 °C for 1 h. After incubation, the solution was centrifuged at 15,890× *g* for 30 min at 4 °C, and the DNA pellet was washed once with cold 70% ethanol and dried in air before being resuspended in 10 mM Tris (pH 8.8).

Viral DNA from immunization material and blood of infected animals was isolated using a modified protocol for total nucleic acid extraction using an automated magnetic bead-based system (Taco-Mini, Genereach, Taichung City, Taiwan). Removal of the DNA from BSL-3 biocontainment, if necessary, was possible after sample DNA was passaged three times on primary PAMs, and hemadsportion assays were performed in triplicate on the third passage to verify inactivation of the infectious virus.

### 2.7. Illumina and Nanopore Sequencing and Data Analysis

Isolated ASFV genomic DNA was submitted for Illumina and Nanopore (ONT) sequencing to MicrobesNG (Birmingham, UK) or sequenced in house. Viruses recovered from high- and low-dose immunized animals were isolated after several limiting dilution passages in PAM. The corresponding viruses were subsequently grown in PAM and NGS sequenced. Libraries were prepared using either the NEBNext Ultra DNA Library Prep Kit (New England Biolabs, Ipswich, MA, USA) or the Nextera XT library prep kit (Illumina, San Diego, CA, USA). DNA libraries were run on Illumina MiSeq or NextSeq platforms as paired end reads (2 × 250 or 2 × 150 bp).

Illumina reads were analyzed as previously described [22]. Briefly, reads were trimmed using trimmomatic software (v0.39), and quality analysis was performed using FastQC software (v0.11.8). Reads were aligned to the parental Arm/07/CBM/c2 wild-type genome using samtools (v1.7), and indexed BAM files were viewed using IGV software (Integrative Genomics Viewer, v2.8.6) using the Arm/07/CBM/c2 wild-type genome as a reference. For variant analysis, GATK (v4.1.9.0) software was used, and variants were mapped to Arm/07/CBM/c2 ORFs using SNPEff software [38] (Version 5.0E 2021-03-09).

Quality control of the long ONT read sequencing data was performed using Nanoplot (https://github.com/wdecoster/NanoPlot, accessed on 23 March 2021). Assembly was performed using the totality of the long ONT reads. For that, we used the software CANU [39] (Version 1.9), an assembler designed for noisy long-read data from PacBio and NanoPore technologies. Only the contigs matching against ASFV were used for the final assembly. For the polishing stage, we used Racon [40] (Version 0.5) and Pilon [41] (Version 1.23) software with the Illumina reads, mapped onto the polished contigs using BWA-MEM [42]. After the whole process, we analyzed the total coverage of the assembly with Illumina and ONT reads. The Illumina reads were mapped with Bowtie2 software [43] (Version 2.3.5.1 64-bit), and the ONT reads with Minimap2. Finally, the genome coverage was calculated from the alignment using the GenomeCoverageBed4 tool [44].

### 2.8. Conventional PCR and Sanger Sequencing

Conventional PCR followed by Sanger sequencing was used for the assessment of the deletion of the MGF region in the 5′ end of the ASFV genome. Several specific primers were designed to: (i) flank the deleted region and (ii) amplify specific genes included in the 5′ end deleted region. The flanking region surrounding the 5′ end deletion was identified between the ASFV_G_ACD_00190 and MGF-360-11L genes, and the primers were designed as follows: 5′-AGATGAGGGGTGTGTGACA-3′ and 5′-CCCGTGATCTGTGCAAAGAG-3′.

Primers corresponding to the genes MGF-110-1L, MGF-360-8L, and MGF-360-9L were designed as follows: 5′-AGGTTGGGAACATTCCATCA-3′ and 5′-CAGAATGCCCACAACACAAC-3′; 5′-GCACATGAGTCGCCACATAC-3′ and 5′-GAGCGTGCCTGAGGAGTATC-3′; and 5′-AGGCCCACTTTAGCATTTCA-3′ and 5′-GCAGCTAGGTGCCAAAGAAC-3′, respectively.

Amplicons were purified from a 1% agarose gel with the Speedtools PCR Clean-Up kit (Biotools, Madrid, Spain). The purified product was subsequently sequenced using the Sanger method (Macrogen, Seoul, Republic of Korea).

### 2.9. Quantitative PCR (qPCR)

For initial confirmation of the deletion of the 5′ MGF region in Arm07∆PolX-GFP, 2 × 10^6^ COS-1 cells were seeded in DMEM supplemented with 2% FBS, and cells were infected at MOI = 2 with either Arm/07/CBM/C2 or the original Arm-∆PolX ASFV virus or mock infected. At 72 hpi, total DNA was harvested from cells using a Speedtools DNA extraction kit (Biotools). DNA was analyzed by qPCR using an ABI PRISM 7900HT SDS real-time PCR detection system (Applied Biosystems, Waltham, MA, USA) with SYBR green master mix (Promega). Gene expression levels were normalized to a housekeeping gene (18S rRNA), and these values were then normalized to the mock-treated sample. The primers used were 5′-GGAGAGGGAGCCTGAGAAAC-3′ and 5′-TCGGGAGTGGGTAATTTGC-3′ for Macaca mulatta 18S rRNA detection, 5′-GTGCACCTATGCAAATCATTG-3′ and 5′-TTTTGTGAAGGTAACTTATTCCTTTG-3′ for ASFV MGF110_1L detection, 5′-GCTCGCTATTTCCATGCTCT-3′ and 5′-TGGTTTTCAAAGACCTTATTCTTACA-3′ for ASFV MGF360_1L detection, 5′-GCAATGATTTTCACCTATTGATAGAC-3′ and 5′-AATCTATGGCGAACTTATACCAAAA-3′ for ASFV MGF360_4L detection, 5′-CAATAATGTGTTTGAACTTCACGA-3′ and 5′-CCACTTTAGCATTTCATTCATGTCT-3′ for ASFV MGF360_9L detection, 5′-AAAAATGATAATGAAACCAATGAATG-3′ and 5′-ATGAGGGCTCTTGCTCAAAC-3′ for ASFV CP204L (p32) detection, and 5′-AATGTTGGGCAGGACGTGTT-3′ and 5′-ACTTACGCTCCCGACTTGC-3′ for ASFV O174L (PolX) detection.

Alternative TaqMan probe-based quantitative PCR (qPCR) assays were performed on ASFV immunization material used in animal experiments as well as clarified blood collected from inoculated piglets in EDTA tubes. qPCR was performed on samples to detect B646L (p72), as described previously [45] with minor modifications [46]. Additional primer and probe sequences used are as follows: MGF110-1L (Forward primer: 5′-CAGTCCCAACAGAACCTACAA-3′; Reverse Primer: 5′-GAGCTAGAGCTCCTGGATCTAA-3′; and Probe: 5′-HEX-AGGTGCACC-ZEN-AGTATTCAAGCTCCT-3′-IABkFQ), MGF360-1L (Forward primer: 5′-AAGCTTTCCTCGCACCTAAC-3′; Reverse primer: 5′-ATCAGCTTTGGGTTGGTTACT-3′; and Probe: 5′-HEX-TTTGGCATA-ZEN-GGTCCTGGTGCACTC-3′-IABkFQ), MGF360-4L (Forward primer: 5′-TCTCTCTAAGGCACGGTCAA-3′; Reverse primer: 5′-ACGTCATGATGTTTCTTGCCT-3′; and Probe: 5′-HEX-ACCTAATTT-ZEN-ATTCCCAGGGCGTGCA-3′-IABkFQ), and MGF360-9L (5′-CCTCCACAGCTTTCACCAA-3′; Reverse primer: 5′-GTGGCATAATGGCCCTATCA-3′; and Probe: 5′-HEX-AACGGCACA-ZEN-AATCTTAACGCGGC-3′-IABkFQ). qPCR was performed using PerfeCTa FastMix II (QuantaBio, Beverly, MA, USA) according to the manufacturer’s instructions with forward/reverse primers and probe at a final concentration of 200 nM in a 20 µL reaction using 5 µL of DNA sample per well. All qPCR reactions were performed in duplicate with extraction and PCR positive and negative controls using a CFX96 Real-Time System (Bio-Rad, Hercules, CA, USA) using the following thermocycling conditions: 95 °C for 5 min, followed by 45 cycles of 95 °C for 10 s and 60 °C for 1 min. Data were analyzed using Bio-Rad CFX Manager (Version 2.3) software using a regression model for Ct determination. Quantification of DNA copy number (CN) for each assay was determined by performing qPCR on serial dilutions of a known quantity of commercially manufactured plasmids (Integrated DNA Technologies, Coralville, IA, USA) containing the respective target sequences to generate a standard curve necessary to convert Ct values to CN/mL.

## 3. Results

### 3.1. Generation of ASFV-Recombinant Virus Arm-ΔPolX by CRISPR-Cas9 from the Virulent Arm/07/CBM/c2 Virus

A modified CRISPR-Cas9 technology in COS-1 cells was used to generate recombinant ASF viruses. Here, the goal was to target and delete the O174L gene from the genome of a virulent ASFV virus, which encodes PolX, a DNA repair polymerase.

**Figure 1:**
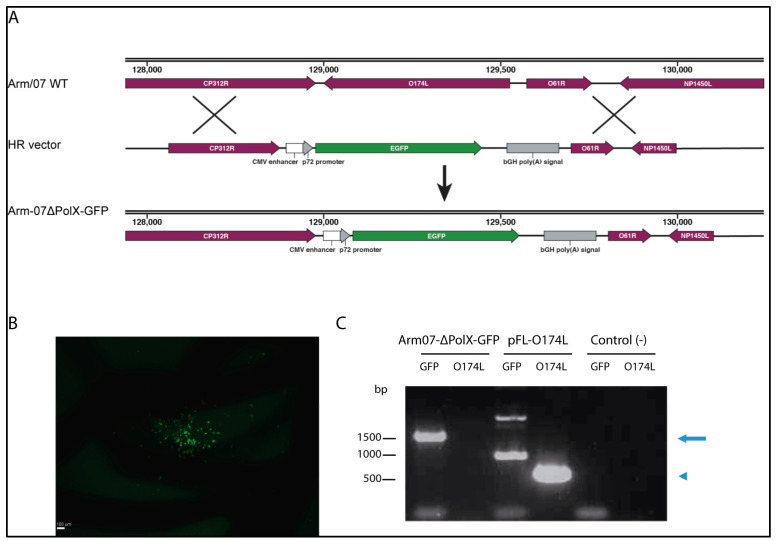
Generation of the recombinant Arm-∆PolX virus. (**A**) Schematic for the generation of Arm-∆PolX. Representation of the original wild-type Arm/07/CBM/c2 genome and the homologous recombination (HR) vector that contains the EGFP gene under the control of the p72 promoter and the flanking regions of the O174L gene (upper two images). The resulting recombinant Arm∆PolX virus is shown in the bottom genome schematic. (**B**) GFP-positive COS-1 cells were identified using fluorescence microscopy, indicating generation of the recombinant Arm-∆PolX virus. (**C**) Specific amplification of either GFP cassette (arrow) or O174L gene (arrowhead) from Arm-ΔPolX or pFL-O174L vector control, by PCR.

The new recombinant ASFV Arm-ΔPolX deletion virus was derived from Arm/07/CBM/c2 [22]. Briefly, COS-1 cells were transfected with two specific vectors coding for: (i) ΔNLS-Cas9 nuclease with specific gRNAs targeting the O174L gene and (ii) the flanking regions of O174L with the target O174L gene replaced with the enhanced GFP (EGFP) reporter gene under the ASFV viral p72 promoter (Figure 1A). Transfected cells were selected with puromycin and then infected with wild-type Arm/07/CBM/c2 to generate the recombinant virus. The resulting recombinant virus was visualized by fluorescence microscopy and isolated by collecting the lysed cultures of GFP-positive COS-1 cells (Figure 1B). Specific PCR amplification confirmed the replacement of the O174L gene by a GFP cassette under the viral p72 promoter (Figure 1C). Analysis of the ASFV Arm-∆PolX genome by Illumina confirmed the absence of reads corresponding to the O174L gene.

### 3.2. The Recombinant Arm-∆PolX Virus Contains an Extra 5′ End Deletion

**Figure 2:**
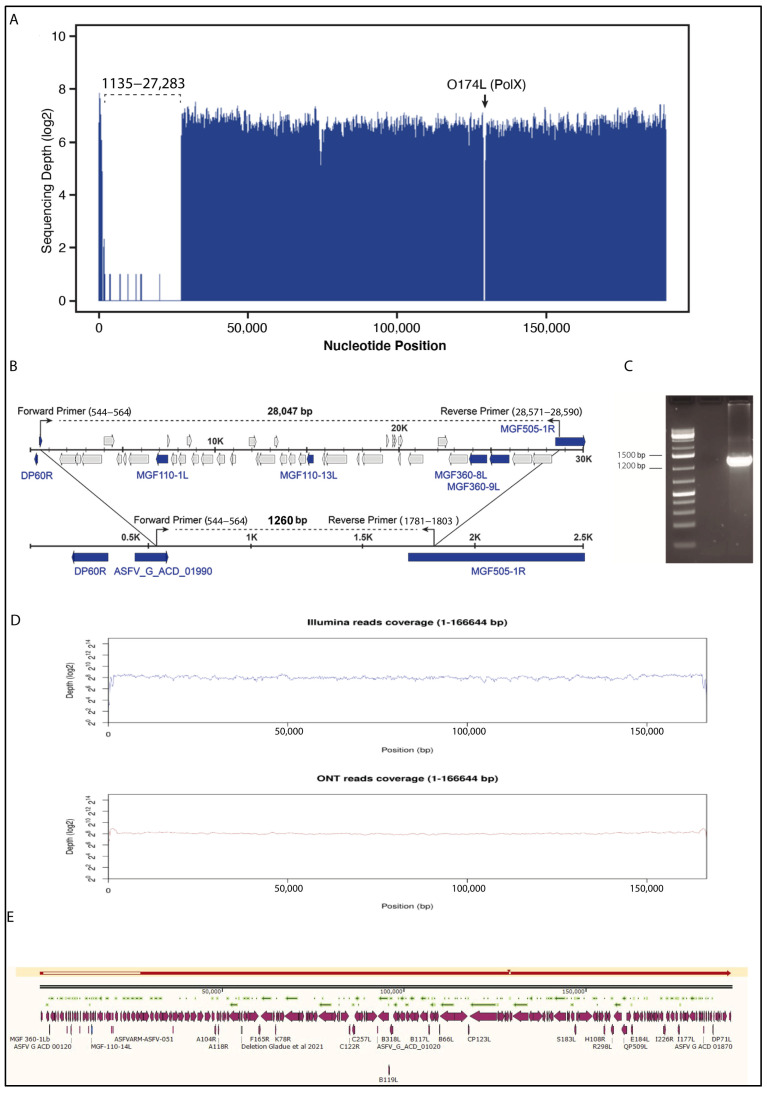
Analysis of the 5′ end deletion in Arm-∆PolX-∆MGF. (**A**) Whole genome coverage plot using Illumina reads of the recombinant Arm-ΔPolX-ΔMGF virus mapped to the ASFV Arm/07/CBM/c2 genome. (**B**) Genomic map of Arm07 showing the putatively deleted region in the 5′ end of the genome. (**C**) PCR amplification of 1,260 bp amplicon confirming the 5′-end GAP. (**D**) Whole genome coverage plot using Illumina (above) or ONT (below) reads of the recombinant Arm-ΔPolX-ΔMGF virus mapped to the de novo assembled genome of Arm-ΔPolX-ΔMGF. (**E**) Pairwise alignment between the Arm/07/CBM/c2 parental genome and Arm-ΔPolX-ΔMGF using Snapgene Software (Version 3.2.1).

Unexpectedly, a coverage plot analysis of NGS data from the Arm-∆PolX recombinant virus revealed a significant drop in coverage corresponding to the 5′ end of the genome, with a low percentage of reads (0.04%) corresponding to nucleotides (nt) 1135 to 27,283 (Figure 2A). To verify that the observed deletion was not a sequencing artifact, we designed specific primers to amplify a region of 1.3 kilobases (kb) that would bridge the predicted deleted region in the 5′ end of the Arm07∆PolX-GFP genome (Figure 2B). Consistent with the NGS data indicating a large 5′ end deletion, we observed a band with the expected length of 1.3 kb (Figure 2C). Sanger sequencing confirmed a deletion of 26 kb in the 5′ end of the Arm-∆PolX genome. The deletion encompasses a total of 41 genes in the Arm07∆PolX-GFP genome, mostly ASFV multigene family (MGF) members: MGF 360-1La, MGF 360-1Lb, MGF 360-2L, KP177R, L83L, L60L, MGF 360-3L, MGF 110-1L, ASFV G ACD 00090, MGF 110-2L, MGF 110-3L, ASFV G ACD 00120, MGF 110-4L, MGF 110-5L-6L, MGF 110-7L, ASFV G ACD 00160, 285L, MGF 100-1R, ASFV G ACD 00190, MGF 110-9L, ASFV G ACD 00210, MGF 110-11L, MGF 110-14L, MGF 110-12L, MGF 110-13La, ASFV G ACD 00270, MGF 360-4L, ASFV G ACD 00300, MGF 360-6L, ASFV G ACD 00320, ASFV G ACD 00330, ASFV G ACD 00350, ASFV G ACD 00360, X69R, MGF 300-1L, MGF 300-2R, MGF 300-4L, MGF 360-8L, MGF 360-9L, MGF 360-10L, and MGF 360-11L (Figure 2B). The 5′ deletion virus with the PolX deletion was designated Arm-ΔPolX-ΔMGF. Unexpectedly, this mutant grew similarly to the wild-type virus in PAM cells but displayed a slightly lower growth kinetic in COS cells (Figure S1).

To determine whether the lost 26 kb fragment in the 5′ end of the genome is caused by a deletion in the genome or a reordering of the genome, we sequenced the entire Arm-ΔPolX-ΔMGF recombinant virus genome using a combination of Illumina short-reads and Oxford Nanopore Technologies (ONT) long-reads. ONT long reads allow for de novo assembly of the viral genome without inference of other reference genomes, allowing for the resolution of highly complex repeated sequences and the identification of genomic rearrangements. The Arm-ΔPolX-ΔMGF ONT long-reads average length was determined to be 1669.3 nucleotides, and the resulting genome sequence was polished with Illumina reads, which are shorter but with a higher fidelity accuracy at the nucleotide level. The resulting viral genome had a total length of 166,644 nucleotides.

To assess the quality of this assembly, we aligned the reads from Arm-ΔPolX-ΔMGF sequencing, both from Illumina and ONT reads, against the generated assembly. Figure 2D shows that coverage with Illumina (blue) and ONT (red) reads is constant, with a read depth of log_2_ 8 in both cases, indicating a high coverage for each base. Pairwise alignment between Arm/07/CBM/c2 parental genome and the assembled Arm-ΔPolX-ΔMGF sequence using Snapgene Software (Version 3.2.1) again showed a 26kb deletion in the 5′ end of the genome that was not due to rearrangement (Figure 2E).

**Figure 3:**
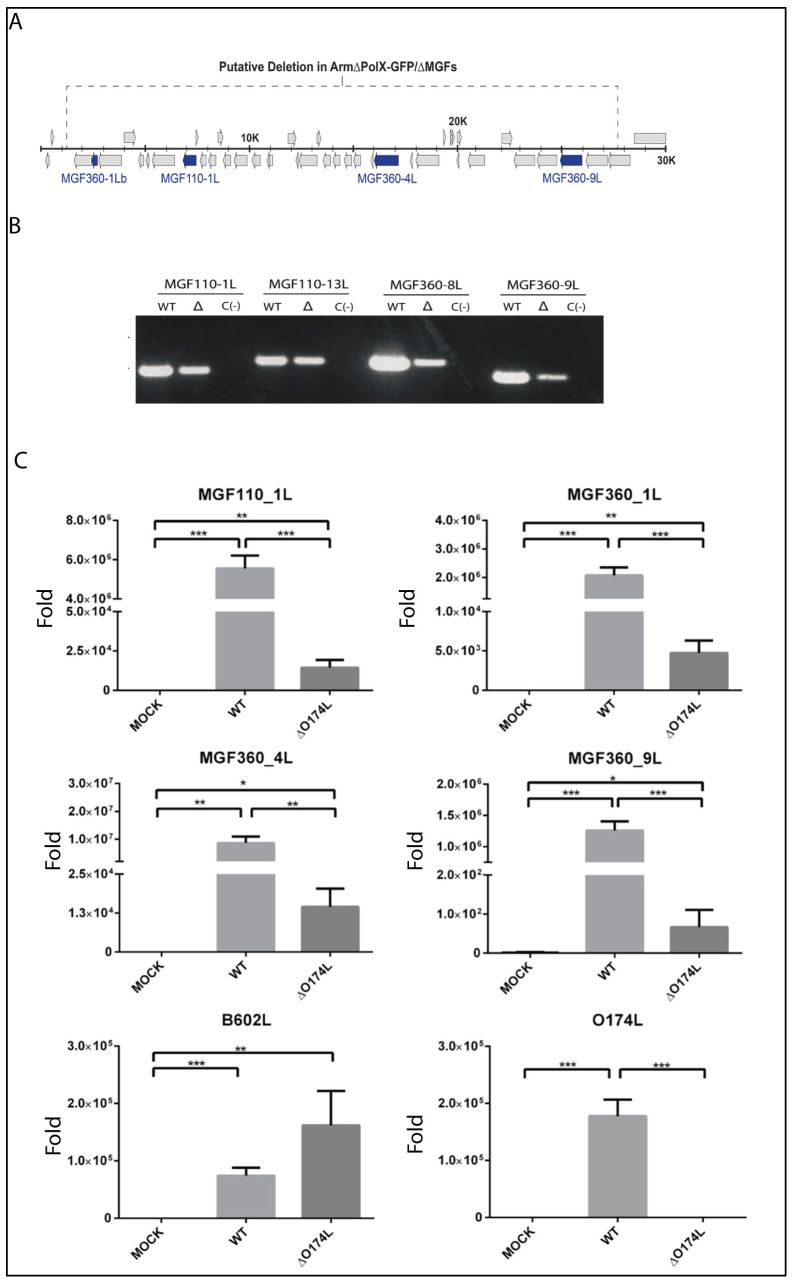
(**A**) Genomic map of Arm/07/CBM/c2 showing the putatively deleted region in the 5′ end of the genome and the four MGF genes targeted for PCR and qPCR analysis (blue). (**B**) PCR amplification of the indicated genes from DNA of wild-type Arm/07/CBM/c2 (WT), Arm-∆PolX (∆), or negative control (C-). (**C**) DNA detection by qPCR of MGF110-1L, MGF360-1L, MGF360-4L, MGF360-9L, B602L, and O174L (PolX) genes in wild-type Arm/07/CBM/c2 and the recombinant virus stock. Significance is denoted as * *p* < 0.05, ** *p* < 0.01, and *** *p* < 0.001.

Despite the 26kb 5′-end deletion in the assembled Arm-ΔPolX-ΔMGF genome, we were able to PCR amplify four of the genes within the deleted 5′ end region: MGF-110-1L, MGF-110-13L, MGF-360-8L, and MGF-360-9L (Figure 3A,B). The identity of the amplified sequences was further confirmed by Sanger sequencing [22], indicating that the virus stock contained two viral populations: (i) one lacking a 26 kb region of MGF genes within the 5′ end of the genome together with the PolX deletion (Arm-∆PolX-∆MGF) and (ii) the other representing a virus with a single PolX deletion only (Arm-∆PolX). To determine the proportion of these two viral populations within the initial virus stock, we used quantitative PCR (qPCR) to quantify the relative presence of several MGF genes in the virus stock compared to the wild-type Arm/07/CBM/c2 virus. The MGF110-1L, MGF360-1L, MGF360-4L, and MGF360-9L genes were found in both the Arm/07/CBM/c2 wild-type and recombinant virus stock by qPCR, but the level of detection of these genes in the recombinant virus stock was around 1000× lower (Figure 3C). A viral gene used as a control (B602L) showed a similar level of detection in both the recombinant virus stock and the wild-type virus, indicating a 1000-fold higher amount of virus containing the double deletion of both the O174L gene and the 26kb 5′ genome segment (i.e., Arm-ΔPolX-ΔMGF) compared to virus possessing the O174L deletion alone (i.e., Arm-ΔPolX).

### 3.3. Immunization with the Mixed Arm-∆PolX/Arm-∆PolX-∆MGF Inoculum Produces Dose-Dependent Clinical Outcomes Related to Viral Virulence

To evaluate the in vivo dynamics of the two viral populations and assess their degree of attenuation, an immunization study was carried out with the mixed inoculum in animals. Two separate groups of piglets were inoculated IM with 10^2^ pfu (low doses) or 10^4^ pfu (high dose) per animal, with five animals per group (Study #1; Table 1). The animals were observed for 14 days for the development of clinical signs consistent with ASF.

**Figure 4:**
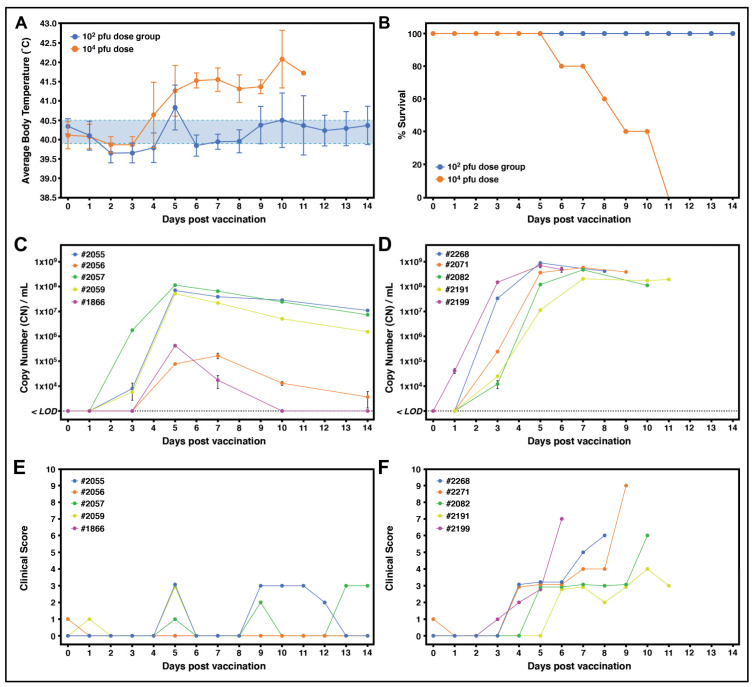
Clinical safety profile study of pigs inoculated with the mixed Arm-∆PolX-/Arm-∆PolX-∆MGF inoculum. (**A**) Average daily temperatures of pigs receiving a 10^2^ (blue) or 10^4^ (orange) pfu dose of the mixed Arm-∆PolX-/Arm-∆PolX-∆MGF inoculum, revealing an increase in average body temperature in pigs receiving the higher dose. (**B**) Survival analysis of piglets receiving a 10^2^ (blue) or 10^4^ (orange) pfu dose of the mixed Arm-∆PolX-/Arm-∆PolX-∆MGF inoculum. (**B**,**D**) Quantitative PCR (qPCR) was performed on pig blood to detect ASFV the B646L (p72) gene in pigs receiving the 10^2^ pfu (**C**) or 10^4^ pfu (**D**) dose of the Arm-∆PolX-/Arm-∆PolX-∆MGF mix. (**E**,**F**) Clinical scores of pigs receiving the 10^2^ pfu (**E**) or 10^4^ pfu (**F**) dose of the Arm-∆PolX-/Arm-∆PolX-∆MGF mix. Total scores were calculated based on several parameters, including fever (0–4), liveliness (0–3), body shape (0–3), respiratory function (0–3), neurological signs (0–3), skin lesions (0–3), ocular/nasal discharge (0–3), and digestive signs (0–3).

All piglets inoculated with the high dose of 10^4^ pfu developed significant fever >40.6 °C between 4- and 6-days post-vaccination (DPV) (Figure 4A and Table S1). Additional clinical signs of ASF, including lethargy, diminished body shape, digestive issues, and inappetence, were observed in all pigs receiving the 10^4^ pfu dose between 5 and 10 DPV (Figure 4F and Table S2). All pigs inoculated with the high dose of 10^4^ pfu died or were humanely euthanized between 6 and 11 DPV (Figure 4B). qPCR performed on blood samples from these pigs indicated that all of them developed ASFV DNAemia with over 10^8^ CN/mL post-inoculation (Figure 4D).

In contrast, piglets inoculated with the low dose of 10^2^ pfu did not succumb to fulminant disease or exhibit overt signs of acute ASF. While 4/5 pigs did develop fevers, the increases in body temperature were generally transient and did not persist for more than 4 days (Figure 4A and Table S1). The combined average body temperatures of pigs receiving the 10^2^ pfu dose increased above normal limits on 5 DPV, but otherwise remained within a normal range (Figure 4A). ASFV p72 DNA was detected in the blood of all pigs receiving the 10^2^ pfu dose; however, the levels were lower compared to pigs receiving the 10^4^ pfu dose, with >10^8^ CN/mL only detected in one pig (#2057) on 5 DPC and with one pig (#1866) fully clearing the infection by the end of this study (Figure 4C). Additionally, viremia/DNAemia showed a decreasing trend in all pigs in the low-dose group by the end of this study on day 14 (Figure 4C). Aside from fever, no other clinical signs indicative of ASF were observed for any of the pigs receiving the 10^2^ pfu dose (Figure 4E and Table S2), in contrast to the high-dose group (Figure 4F), and all animals survived to the end of this study (Figure 4B). Overall, these results indicate that the Arm-∆PolX-/Arm-∆PolX-∆MGF mix results in lethal ASF disease at a 10^4^ pfu dose but is attenuated at a lower dose of 10^2^ pfu with 100% survival under the experimental conditions used.

**Figure 5:**
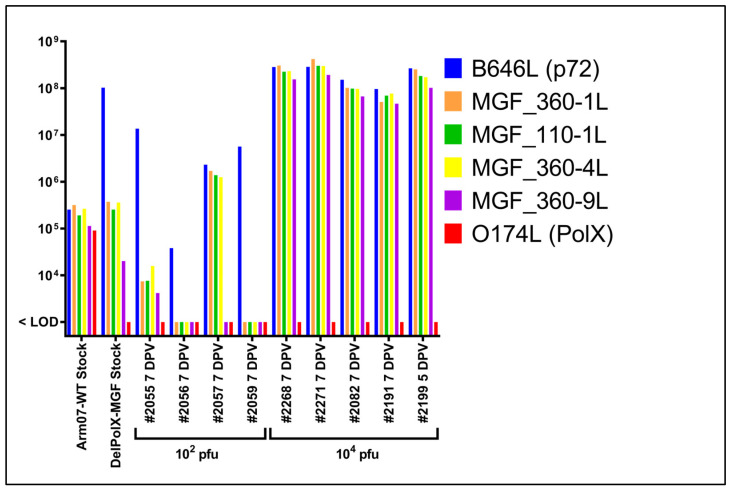
Quantitative PCR (qPCR) to detect various ASFV genes. qPCR was performed to detect the B646L (p72), MGF360-1L, MGF110-1L, MGF360-4L, MGF360-9L, and O174L (PolX) genes. Samples tested are: (i) wild-type Arm/07/CBM/c2 and the mixed Arm-∆PolX/Arm-∆PolX-ΔMGF virus stock used in the in vivo studies; (ii) blood collected on 7 dpv from pigs receiving the 10^2^ pfu dose of the virus stock (Study #1); and (iii) blood collected on 7 DPV (or 5 DPV; pig #2199) from pigs receiving the 10^4^ pfu dose of the virus stock (Study #1). The relative amount of the B646L (p72) gene in relation to MGF genes provides insight into the relative amount of the single gene-deleted Arm-∆PolX virus versus the double-deleted Arm-∆PolX-∆MGF virus present in the respective pigs’ blood at the indicated time point.

To assess whether the relative proportion of Arm-ΔPolX and Arm-ΔPolX-ΔMGF vaccine virus subpopulations shifted following inoculation, qPCR was performed to discriminate between the two recombinant viruses during viremia in the immunized animals. We used qPCR to quantify the amount of the ASFV genes B646L (p72), O174L (PolX), and the MGF members MGF360-1L, MGF110-1L, MGF360-4L, and MGF360-9L. The O174L gene is absent in both the Arm-ΔPolX and the Arm-ΔPolX-ΔMGF viruses, while the MGF genes are absent in the Arm-∆PolX-∆MGF virus (Figure 5).

Interestingly, in all pigs infected with the 10^4^ pfu high dose of virus stock, roughly equivalent levels of the p72 and MGF genes were detected at later time points post-immunization, indicating the Arm-∆PolX virus outcompeted the Arm-∆PolX-∆MGF virus in the high-dose group. In contrast, in pigs receiving the 10^2^ pfu low dose, two of the animals (pigs #2056 and #2059) were negative for the MGF genes despite the detection of the p72 gene, indicating that only the double-deleted Arm-∆PolX-∆MGF virus was present in these pigs at 7 DPV. Three out of 4 MGF genes (except for MGF360-9L) were detected in pig #2057 at a level equivalent to the p72 gene, indicating that the single Arm-∆PolX-GFP virus was the dominant virus population in this pig. All four MGF genes were detected in the low-dose vaccinated pig #2055 at an approximately 1000-fold lower rate than the p72 gene, similar to the proportion observed in the inoculum. These results suggest higher attenuation of the double-deleted Arm-∆PolX-∆MGF mutant, since three out of four pigs who survived vaccination had qPCR results indicative of the presence of the double-deleted Arm-ΔPolX-ΔMGF virus at 7 DPC.

### 3.4. Genetic Characterization of Virus Obtained from Immunized Pigs

To determine the genome stability of either Arm-ΔPolX or Arm-ΔPolX-ΔMGF in vivo, we analyzed the whole ASFV genome from plasma samples of pigs #2268 and #2271, which both received the 10^4^ high dose. Variant calling analysis compared to the Arm/07/CBM/c2 reference sequence revealed that no single nucleotide polymorphisms (SNPs) or indels were detected in virus collected from pig #2268, and only two SNPs were detected in virus collected from pig #2271 (Table 2), indicating that the absence of PolX did not significantly increase the number of DNA mutations during in vivo infection.

According to what we previously observed (Figure 5), a whole genome coverage plot using Illumina reads indicates the presence of 5′ MGF genes in the virus recovered from animal #2268 (Figure S2).

### 3.5. Vaccination with Purified Arm-ΔPolX or Arm-ΔPolX-ΔMGF Viruses Confirms That PolX Deletion Alone Does Not Lead to In Vivo Attenuation

NGS sequencing confirmed the presence of the ΔPolX and ΔPolX-ΔMGF deletions, as well as the absence of significant off-target mutations in the purified viruses (Figure S3 and Tables S3 and S4). Subsequently, the purified Arm-ΔPolX and Arm-ΔPolX-ΔMGF viruses were used for a safety study in animals (Study #2; Table 1). Here, we first analyzed the attenuation of the two recombinant viruses and then their ability to induce protection against a virulent challenge.

**Figure 6:**
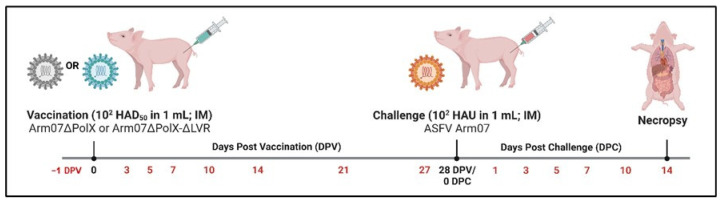
Overview of vaccination and challenge experiment. Piglets were divided into three groups of 6 animals each. Groups 1 and 2 were vaccinated IM with 10^2^ HAD50 Arm-ΔPolX or Arm-ΔPolX-ΔMGF, with group 3 serving as unvaccinated controls. After 28 days, the surviving vaccinated animals and controls were challenged IM with 10^2^ HAD50 Arm/07/CBM/c2. Necropsies were performed at the time of animal death or were scheduled for 14 days post-challenge for any animals surviving the challenge. Scheduled blood collections were performed on days listed in red.

**Figure 7:**
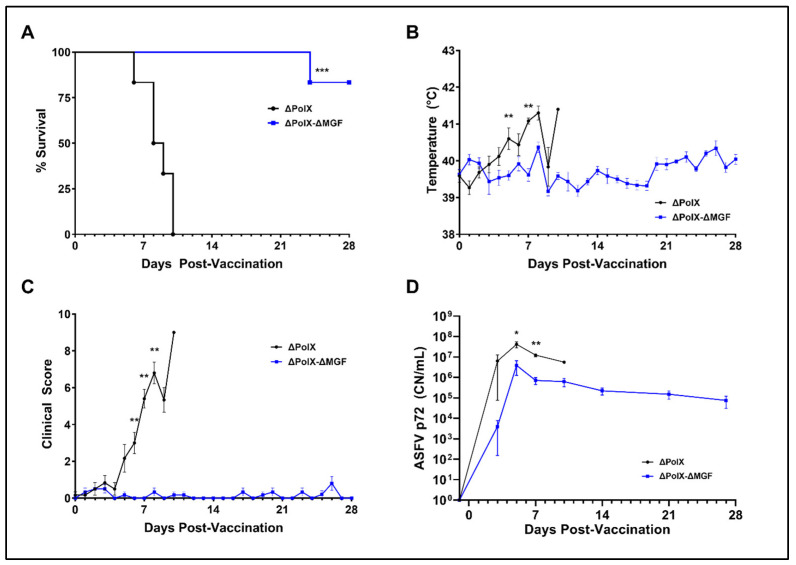
Arm-ΔPolX-ΔMGF is attenuated in vivo while Arm-ΔPolX retains its virulence. (**A**) Survival curves for Arm-ΔPolX and Arm-ΔPolX-ΔMGF vaccine groups. Curves were compared using the Mantel–Cox test. (**B**) Daily body temperatures for Arm-ΔPolX and Arm-ΔPolX-ΔMGF vaccine groups. (**C**) Daily clinical scores for Arm-ΔPolX and Arm-ΔPolX-ΔMGF vaccine groups. (**D**) qPCR values for Arm-ΔPolX and Arm-ΔPolX-ΔMGF groups from vaccination to challenge. Graphs were compared using multiple Mann–Whitney tests, and significant differences were noted at specific timepoints. For all panels, significance is denoted as * *p* < 0.05, ** *p* < 0.01, and *** *p* < 0.001.

In the animal study #2 (Figure 6), markedly different clinical outcomes were seen after intramuscular (IM) vaccination with 10^2^ HAD50 of either Arm-ΔPolX or Arm-ΔPolX-ΔMGF viruses. All 6 animals that received the 10^2^ dose of Arm-ΔPolX died with acute ASF by 10 DPV, whereas 5/6 animals inoculated with 10^2^ of the Arm-ΔPolX-ΔMGF lived until virulent ASF challenge at 28 DPV (Figure 7A). Body temperatures for the Arm-ΔPolX group trended to be higher than for the Arm-ΔPolX-ΔMGF group, with a statistically significant difference at 5 and 7 DPV (Figure 7B). Similarly, clinical scores for the Arm-ΔPolX group showed a rapid increase beginning at 5 DPV, which persisted until all animals died or were humanely euthanized, whereas animals receiving the Arm-ΔPolX-ΔMGF virus developed little to no clinical symptoms. Statistically significant elevations in clinical score were observed for the Arm-ΔPolX group at 6, 7, and 8 DPV (Figure 7C). ASFV p72-specific qPCR values in the Arm-ΔPolX animals were consistent with the observed lack of attenuation for the Arm-ΔPolX virus. All animals that received the Arm-ΔPolX had detectable levels of ASFV DNA in whole blood by 5 DPV, with maximum values approaching 10^8^ CN/mL. ASFV DNA levels were significantly higher in the Arm-ΔPolX group compared to the Arm-ΔPolX-ΔMGF group at 5 and 7 DPV (Figure 7D). In the Arm-ΔPolX-ΔMGF group, 5/6 animals had detectable levels of ASFV DNA in blood at 5 DPV, and all animals that survived vaccination maintained detectable levels of ASFV viral DNA until challenge at 28 DPV, albeit with a trend toward decreasing concentrations of viral DNA over time (Figure 7D). One animal in the Arm-ΔPolX-ΔMGF group (#291) did not develop detectable levels of ASFV DNA at any time point post-vaccination up to the time of challenge. These results indicate that a deletion of PolX by itself does not lead to attenuation, while the presence of the additional deletion at the 5′ end of the genome does produce attenuation at the 10^2^-immunization dose.

### 3.6. Immunization with Arm-ΔPolX-ΔMGF Does Not Induce Protection against Virulent Parental Virus Challenge

**Figure 8:**
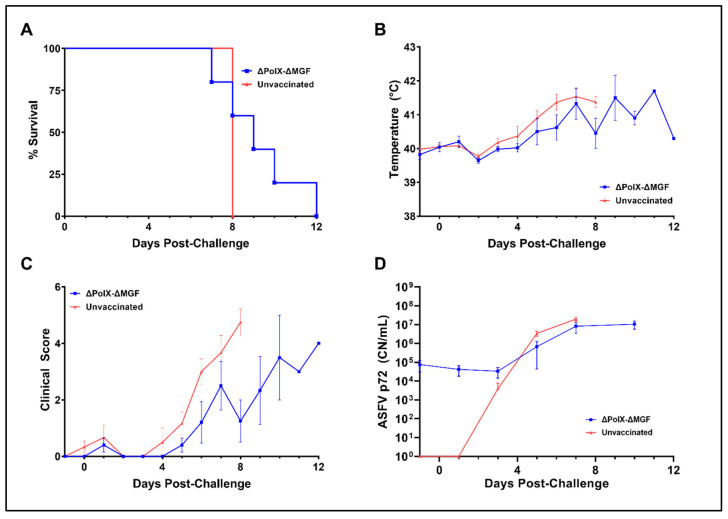
Immunization with Arm-ΔPolX-ΔMGF did not confer protection against a homologous virulent challenge. (**A**) Survival curves for Arm07ΔPolX-ΔMFG vaccinated and unvaccinated animals. Curves were compared using the Mantel–Cox test. (**B**) Daily body temperatures for Arm-ΔPolX-ΔMGF and unvaccinated animals. (**C**) Daily clinical scores for Arm-ΔPolX-ΔMGF and unvaccinated animals. (**D**) qPCR values for Arm-ΔPolX-ΔMGF and unvaccinated animals. All graphs were compared using multiple Mann–Whitney tests but no significant differences at specific timepoints were found.

To evaluate if immunization with Arm-ΔPolX-ΔMGF was able to induce protective immunity, five animals that survived immunization (5/6) were challenged with 10^2^ HAD50 of the virulent parental Arm/07/CBM/c2 ASFV strain, with 6 additional animals serving as unvaccinated controls. A similar clinical course of disease was observed in both groups after challenge (Figure 8). All unvaccinated animals died at 8 DPC, whereas deaths in the Arm-ΔPolX-ΔMGF group were staggered and occurred from 7–12 DPC; however, no statistically significant difference was present between both groups (Figure 8A). Animals in both groups were clinically healthy prior to, and at the time of, virulent challenge, then subsequently developed clinical symptoms characteristic of acute ASF. No significant differences in temperature or clinical scores were observed between unvaccinated and Arm-ΔPolX-ΔMGF groups at any timepoint (Figure 8B,C). In the Arm-ΔPolX-ΔMGF vaccinated group, 4/5 animals were qPCR positive for ASFV DNA prior to challenge, and the negative animal became positive by 5 DPC (Figure 8D). Unvaccinated animals began to show detectable levels of ASFV DNA in blood at 3DPC, with all 6 animals showing qPCR positivity by day 7. No significant differences in viral DNA levels were seen between groups at any timepoint following the appearance of viremia in unvaccinated animals at 3 DPC. Since all animals showed evident signs, symptoms, and gross pathological lesions typical of acute ASF and there was no difference in clinical scores, survival time, or viral DNA levels after challenge between vaccinated animals and mock controls, histopathological evaluation of tissues after challenge was not performed.

## 4. Discussion

In this study, we generated a novel ASFV recombinant virus lacking the O174L gene encoding the viral polymerase PolX in COS-1 cells. While porcine PBMCs or macrophages have been used for LAV generation [4,5,13,14,47], the use of primary cells for vaccine virus production lacks reproducibility, is expensive, and raises significant concerns regarding pathogen contamination and animal welfare. The COS-1 cell line is permissive for ASFV infection and could potentially be used for production and scale-up of an ASF LAV candidate [6,8,11,36]. However, the use of cell lines derived from animal species that are not natural hosts for ASFV might promote genetic alterations (e.g., deletions) in the ASF genome, as seen during the generation of the present recombinant virus. The appearance of large viral genome deletions and the presence of multiple viral subpopulations have previously been also observed with wild-type ASFVs, which were passaged in Vero cells [48,49] or CV1 [50] cell lines. Interestingly, genetic alterations on the 5′ end of the genome similar to those reported here have also been reported when a recombinant LAV candidate was grown in a porcine fetal kidney cell line [51]. The causes of these deletions, and specifically the deletion that occurred during the generation and/or purification of the Arm-∆PolX mutant, are unknown. However, it could be pointed to errors during viral DNA replication driving aberrant homologous recombination as a hypothesis. The strong homology and sequence identity between MGF genes would favor the occurrence of deletions specifically in this area of the ASFV genome. This off-target 5′ end deletion is not unprecedented, as the ends of the ASFV genome, particularly the 5′ end, are frequently deleted upon passage in cell lines, but not in primary porcine macrophages [48,49,51,52]. MGF members are known to play important roles in host immune response modulation but are dispensable for in vitro growth [9,23,24]. Therefore, we hypothesized that a recombinant ASFV virus harboring this extra-deletion on the 5′ end would confer further attenuation in vivo.

Viral genetic stability is a key issue involved in the generation of recombinant ASFVs in cell lines. Particular attention must be paid to unexpected off-target deletions/insertions/duplications that may arise during the generation of a recombinant virus prototype. Here, we have developed an NGS analysis approach for the detailed genetic characterization of our recombinant prototype virus, Arm-∆PolX. Although minor genetic variations such as indels or SNPs were not detected, an unexpected deletion in the 5′ end of the genome was identified, encompassing 26 kb and more than 40 viral genes, some of which have been attributed to play a role in host immune response modulation [9,24,25,26]. Analysis of the original stock by conventional PCR, coupled with Sanger sequencing and qPCR quantification, allowed us to determine that the virus stock was composed of two viral subpopulations—a major population containing deletions of both the PolX gene and the 26 kb 5′ genome fragment (Arm-∆PolX-∆MGF), and a second minor population possessing only the single PolX gene deletion (Arm-∆PolX). Different virus population dynamics were observed in pigs inoculated with this original mixed virus stock at low and high doses. Animals that were inoculated with the low dose (10^2^ pfu) showed dominant replication of the Arm-∆PolX-∆MGF virus, while in animals that were inoculated with the high dose (10^4^ pfu), the Arm-∆PolX virus became the dominant population, despite this variant being a low proportion (1:1000) of the inoculum. This indicates a superior fitness of the single-gene deletion mutant Arm-∆PolX over the double gene deletion mutant Arm-∆PolX-∆MGF in vivo. We hypothesize that this difference in fitness is due to the ability of the MGF genes, which are present in the single gene deletion mutant Arm-∆PolX, to modulate the host immune response [9,24,25,26].

All animals inoculated with the high dose of virus stock died between 6 and 11 DPV, suggesting a correlation between high virulence and mortality and the presence of the single gene-deleted ∆PolX virus. This was confirmed by separately isolating and purifying the Arm-ΔPolX and Arm-ΔPolX-ΔMGF ASFVs and inoculating pure virus stocks into piglets as part of the second animal experiment. All animals that received purified Arm-ΔPolX succumbed to a virus infection characteristic of virulent ASFV. In contrast, 5/6 animals that received the double-deletion Arm-ΔPolX-ΔMGF virus alone survived vaccination, indicating reduced virulence for this double-deleted recombinant virus. The marker used to replace O174L in both prototypes, GFP, has been used in other LAV prototypes previously [11] and has no relevance to ASFV virulence. However, despite attenuation, none of the animals receiving Arm-ΔPolX-ΔMGF were protected against challenge with the virulent parental ASFV strain, indicating the attenuated Arm-ΔPolX-ΔMGF virus does not induce protective immunity. These results suggest that, although PolX may have a relevant function in vitro in maintaining the integrity of the viral DNA during viral replication within the oxidative environment of the macrophage [21], this was not clearly evident in vivo; we did not find mutations or genomic rearrangements in the single gene-deleted ΔPolX viruses isolated from pigs at 7 DPV. Importantly, deletion of the PolX gene alone is insufficient to attenuate the virus, as evidenced by the mortality observed in animals infected with the single gene-deleted Arm-ΔPolX virus. These data were also recently confirmed with another genotype II ASFV virus, Georgia 2010, where the ∆O174L ASFV mutant presented a clinical form of the disease that is indistinguishable from that induced by the parental virulent strain [53]. In contrast, pigs harboring the double gene deletion mutant Arm-∆PolX/∆MGF at 7 DPV did not show clinical signs, indicating attenuation of the Arm-ΔPolX-ΔMGF virus.

The ASFV MGF genes encompass five different gene families containing around 47 individual genes [54]. Although the functions of a few individual proteins encoded by the MGFs have been described [24,29,55,56], the functions of many still remain unknown. Certain MGF elements are absent from the naturally attenuated strains NH/P68 and OURT88/3, which both confer protection against homologous and heterologous challenge [6,57]. Accordingly, attempts to generate LAVs derived from virulent strains with targeted deletions of these MGF genes, mainly within the MGF360 and MGF505 families, have been described [5,9,12]. Most of the MGF360 and MGF505 genes are present in our recombinant Arm-ΔPolX-ΔMGF virus, indicating the existence of new putative target genes for the development of novel LAVs.

Previously, it has been described that after the adaptation of the LAV G-ΔI117L to grow in the PIPEC cell line, around 10 kb from the 5′ arm of the genome was deleted, although the new recombinant G-ΔI177L/ΔMGF maintained the same complete attenuation phenotype as the parental, as well as protection against virulent challenge. This deletion encompassed 8 genes that were deleted in our LAV prototype: MGF 360-6L, X69R, MGF 300-1L, MGF 300-2R, MGF 300-4L, MGF 360-8L, MGF 360-9L, and MGF 360-10L [51]. Hence, these genes seem to be lost during adaptation of cell line growth, and their potential role in attenuation could not be excluded. In addition, the function of other genes also deleted in the Arm-ΔPolX-ΔMGF prototype has been described. Among them, some have been directly implicated in virulence, such as MGF 110-9L [24] or MGF 360-10L [30,34], while in others, it has been ruled out, as in the case of MGF 360-1L [31] or MGF 110-5L-6L [32].

Deletion of MGF family genes in Arm-ΔPolX-ΔMGF occurring during generation or growth of the recombinant virus in COS-1 cells leads to attenuation of the recombinant virus if compared to Arm-ΔPolX. However, their deletion does not lead to protection against virulent challenge. One possible explanation is that the in vivo growth of the Arm-ΔPolX-ΔMGF prototype does not trigger an effective immune response due to the low dose used. Indeed, previous work has shown that particular prototypes fail to protect with immunizations at low doses, such as 10^3^, but are effective in protection at higher doses, such as 10^6^ [8], although in some cases, they may carry safety concerns. Since immunization with Arm-ΔPolX-ΔMGF was performed at the dose of 10^2^, it would not be ruled out that at higher doses protection against virulent challenges could be achieved, although it would remain to be determined whether the prototype under these conditions remains completely safe.

## 5. Conclusions

In conclusion, our results demonstrate that deletion of the PolX gene alone in a prototype genotype II ASFV is not sufficient to abrogate ASFV virulence in vivo. Amplification of the single gene deletion Arm-ΔPolX-virus in COS-1 cells led to an additional large 5′ end deletion encompassing multiple MGF genes, which did result in attenuation in vivo. However, immunization with the Arm-ΔPolX-ΔMGF virus did not induce protection against challenge with the virulent parental ASFV strain. Additional studies will be required to evaluate the phenomenon involved in the establishment of off-target deletions at the 5′ end of the ASFV genome during the generation of a recombinant virus by homologous recombination or via CRISPR/Cas9 technology and to determine which specific MGF element deletions are associated with an attenuated ASFV phenotype of the double-deletion mutant Arm-ΔPolX-ΔMGF.

## Figures and Tables

**Table 1:** Outline of the in vivo study designs.

Study	Pig ID	Vaccine Virus	Immunization Dose	Challenge Virus	Challenge Virus Dose
Study #1 Safety Study	2055	Mixed population Arm-∆PolX/Arm-∆PolX-∆MGF	10^2^ pfu intramuscular	None	Not applicable
	2056				
	2057				
	2059				
	1866				
	2268		10^4^ pfu intramuscular		
	2271				
	2082				
	2191				
	2199				
Study #2 Safety and efficacy Study	292	Purified Arm-∆PolX	10^2^ pfu intramuscular	Not applicable (All animals died prior to challenge)	Not applicable
	294				
	295				
	298				
	300				
	302				
	291	Purified Arm-∆PolX-∆MGF	10^2^ pfu intramuscular	Arm/07	10^2^ HAD50 intramuscular
	293				
	296				
	297				
	299				
	301	Unvaccinated	Not applicable	Arm/07	10^2^ HAD50 intra-muscular
	304				
	305				
	308				
	309				
	311				

**Table 2:** Illumina sequencing statistics and variant analysis of virus recovered from vaccinated animals #2271 (7 DPV) and #2268 (5 DPV) compared to Arm/07/CBM/c2 genome.

Vaccinated Animal	Mean Coverage	SNP	Indels
#2268	85.7	0	0
#2271	101.2	2	0

## Data Availability

Data are available on request due to restrictions.

## Supplementary Materials

The following supporting information can be downloaded at: https://www.mdpi.com/article/10.3390/vaccines12101125/s1, Figure S1: Arm/07/CBM/c2 and Arm-∆PolX-∆MGF in vitro growth in PAM or COS-1 cells; Figure S2: Whole genome coverage plot using Illumina reads of the virus recovered from animal #2268 mapped to the ASFV Arm/07/CBM/c2 genome; Figure S3: Whole genome coverage plots using Illumina reads of the isolated viruses Arm-ΔPolX (A) and Arm-ΔPolX-ΔMGF (B) mapped to the ASFV Arm/07/CBM/c2 genome; Table S1: Daily body temperatures (°C) of pigs in the initial in vivo safety Study #1. Red indicates temperatures considered fever; Table S2: Clinical sign scores of pigs in the initial in vivo safety Study #1. Red indicates high clinical sign values; Table S3: Illumina sequencing statistics and variant analysis of isolated viruses Arm-ΔPolX mapped to Arm/07/CBM/c2 genome; Table S4: Illumina sequencing statistics and variant analysis of isolated viruses Arm-ΔPolX and Arm-ΔPolX-ΔMGF mapped to Arm/07/CBM/c2 genome.
