# Supplementary material for: Generation and Genetic Stability of a PolX and 5′ MGF-Deficient African Swine Fever Virus Mutant for Vaccine Development
Source: Vaccines (Basel). 2024 Sep 30;12(10):1125. doi: 10.3390/vaccines12101125 (PMC11511218; doi:10.3390/vaccines12101125)
Supplement: Supplementary file 1 [file vaccines-12-01125-s001.zip › vaccines-3197377-supplementary.pdf]

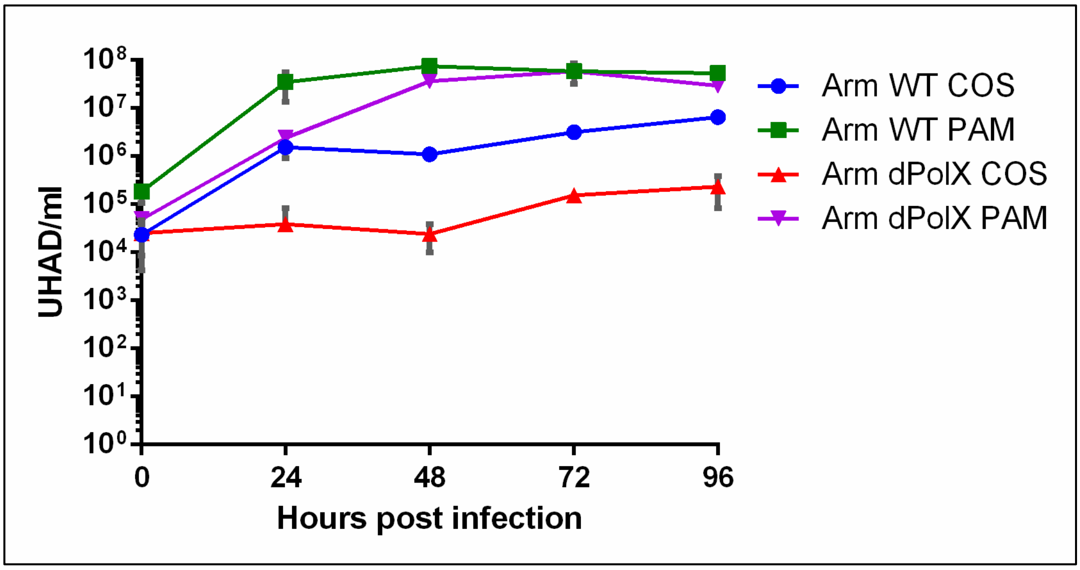

Figure S1. Arm/07/CBM/c2 and Arm-ΔPolX-ΔMGF *in vitro* growth in PAM or COS-1 cells. PAM or COS-1 cells were infected with either Arm/07/CBM/c2 WT or Arm-ΔPolX-ΔMGF at MOI= 2. At the indicated times post infection, total virus was recovered and titrated by HAD assay as indicated in Materials & Methods.

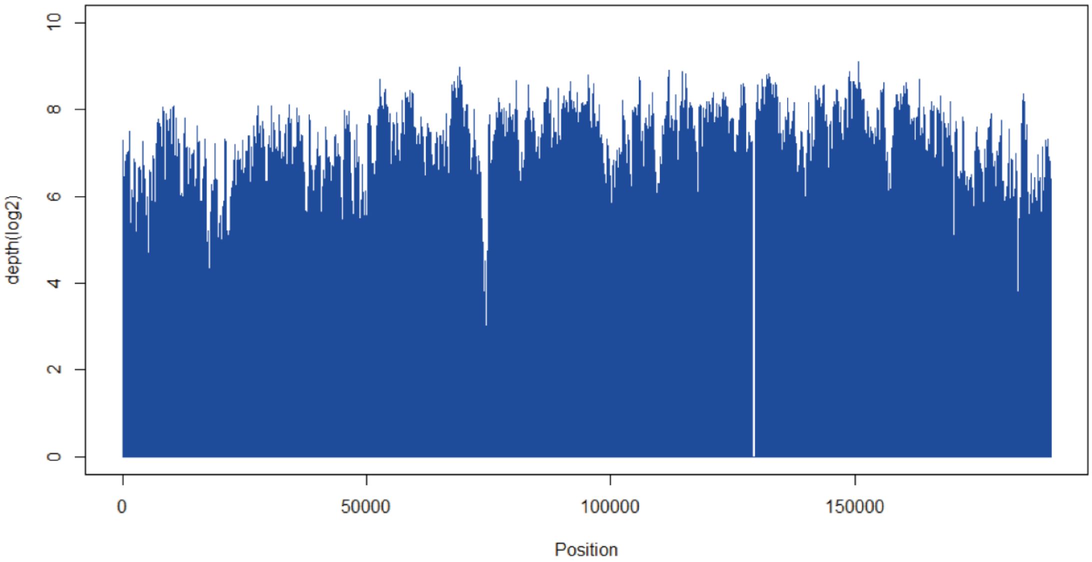

Figure S2. Whole genome coverage plot using Illumina reads of the virus recovered from animal #2268 mapped to the ASFV Arm/07/CBM/c2 genome.

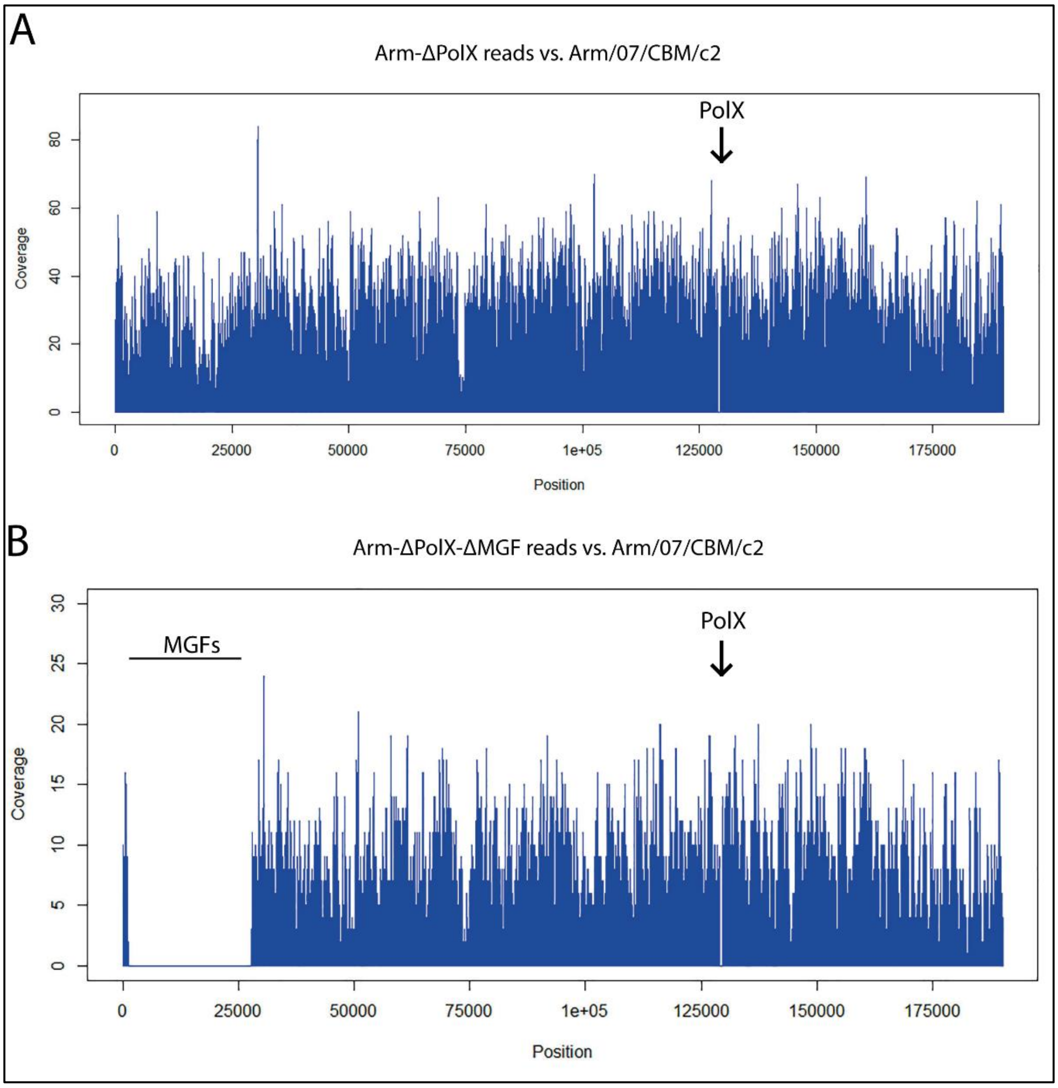

Figure S3. Whole genome coverage plots using Illumina reads of the isolated viruses Arm-ΔPolX (A) and Arm-ΔPolX-ΔMGF (B) mapped to the ASFV Arm/07/CBM/c2 genome.

Table S1. Daily body temperatures (˚C) of pigs in the initial *in vivo* safety Study #1.

			Day Post Vaccination														
**Virus Administered**	**Dose**	**Pig ID**	**0**	**1**	**2**	**3**	**4**	**5**	**6**	**7**	**8**	**9**	**10**	**11**	**12**	**13**	**14**
Arm07ΔPolX-GFP/ΔMGF	10^2 pfu	2055	40.2	39.8	39.5	39.8	39.7	41.4<br>40.6	39.9<br>39.9	39.9<br>40.0	40.2<br>40.6	41.1<br>41.1	41.7<br>41.7	41.7<br>40.9	40.8<br>41.0	40.3<br>40.9	40.2
		2056	40.6	40.1	39.4	39.7	39.4	40.4<br>40.1	40.3<br>40.3	40.1<br>40.3	41.1<br>39.8	40.0<br>40.2	40.4<br>40.2	39.9<br>40.3	40.2<br>40.7	40.3<br>40.5	40.2
		2057	40.1	39.7	39.5	39.8	40.4	40.7<br>40.6	39.8<br>40.3	40.1<br>40.4	40.2<br>40.8	40.7<br>40.4	40.3<br>40.6	40.2<br>40.8	40.4<br>40.9	41.0<br>40.9	41.2
		2059	40.3	40.6	39.8	39.9	39.6	41.5<br>41.0	39.6<br>40.4	39.6<br>40.3	39.7<br>40.1	40.0<br>40.6	40.2<br>39.9	40.1<br>40.1	39.8<br>40.1	39.8<br>40.6	39.8
		1866	40.4	40.3	40.1	39.9	39.7	40.2<br>40.1	39.6<br>40.1	40.0<br>40.1	39.6<br>39.7	40.1<br>40.2	39.9<br>39.9	39.9<br>40.0	39.9<br>40.4	40.1<br>40.1	40.4
	10^4 pfu	2268	39.9	39.6	39.6	40.3	41.4	41.3<br>40.8	41.7<br>41.3	41.9<br>41.9	41.7	-	-	-	-	-	-
		2271	40.6	40.5	40.5	40.2	41.4	41.1<br>41.0	41.3<br>41.3	41.4<br>42.1	41.1<br>42.1	41.5	-	-	-	-	-
		2082	40.2	40.0	40.0	39.9	39.6	41.3<br>41.2	41.5<br>42.1	41.6<br>41.8	41.5<br>41.8	41.2<br>42.5	42.6	-	-	-	-
		2191	40.1	40.2	40.2	39.8	39.9	40.4<br>40.0	41.8<br>41.7	41.2<br>41.8	40.9<br>41.9	41.4<br>42.0	41.6<br>41.7	41.7	-	-	-
		2199	39.7	40.1	40.1	41.0<br>40.9	40.9	42.3<br>41.8	41.4	-	-	-	-	-	-	-	-

Table S2. Clinical sign scores of pigs in the initial *in vivo* safety Study #1.

Virus Administered	Dose	Pig ID	Day Post Vaccination 0	1	2	3	4	5	6	7	8	9	10	11	12	13	14
Arm07ΔPolX-GFP/ΔMGF	10^2 pfu	2055	0	0	0	0	0	3<br>1	0<br>0	0<br>0	0	3<br>3	3<br>3	3<br>2	2<br>2	0<br>2	0
		2056	1	0	0	0	0	0<br>0	0<br>0	0<br>0	0	0<br>0	0<br>0	0<br>0	0<br>1	0<br>1	0
		2057	0	0	0	0	0	1<br>1	0<br>0	0<br>0	0	2<br>3	0<br>1	0<br>1	0<br>2	3<br>3	3
		2059	0	1	0	0	0	3<br>1	0<br>0	0<br>0	0	0<br>1	0<br>0	0<br>0	0<br>0	0<br>1	0
		1866	0	0	0	0	0	0<br>0	0<br>0	0<br>0	0	0<br>0	0<br>0	0<br>0	0<br>0	0<br>0	0
	10^4 pfu	2268	0	0	0	0	3	3<br>2	3<br>3	5<br>4	6	-	-	-	-	-	-
		2271	1	0	0	0	3	3<br>3	3<br>3	4<br>4	4	9	-	-	-	-	-
		2082	0	0	0	0	0	3<br>3	3<br>3	3<br>3	3	3<br>4	6	-	-	-	-
		2191	0	0	0	0	0	0<br>0	3<br>3	3<br>3	2	3<br>3	4<br>3	3	-	-	-
		2199	0	0	0	1	2	3<br>3	7	-	-	-	-	-	-	-	-

Table S3. Illumina sequencing statistics and variant analysis of isolated viruses Arm-ΔPolX mapped to Arm/07/CBM/c2 genome.

Position	Variant	Gene	Effect
14005	ACC → AC/A	MGF 110-14L	Frameshift. Fusion protein with MGF 110-11L
21575	C → CG	Intergenic region	-

Table S4. Illumina sequencing statistics and variant analysis of isolated viruses Arm-ΔPolX and Arm-ΔPolX-ΔMGF mapped to Arm/07/CBM/c2 genome.

Position	Variant	Gene	Effect
39769	AT → A	Intergenic region	-
80844	G → GA	M448R	Frameshift. Lys203STOP.
106814	C → A	B117L	Gly74Val
185703	A → G	Intergenic región	-
186119	A → T	MGF 360-19R	Lys34STOP
